# Elucidation of the Lipid Composition of Hemp (*Cannabis sativa* L.) Products by Means of Gas Chromatography and Ultra-High Performance Liquid Chromatography Coupled to Mass Spectrometry Detection

**DOI:** 10.3390/molecules27103358

**Published:** 2022-05-23

**Authors:** Paola Arena, Francesca Rigano, Paolo Guarnaccia, Paola Dugo, Luigi Mondello, Emanuela Trovato

**Affiliations:** 1 Department of Chemical, Biological, Pharmaceutical and Environmental Sciences, University of Messina, 98168 Messina, Italy; paola.arena@unime.it (P.A.); pdugo@unime.it (P.D.); lmondello@unime.it (L.M.); emanuela.trovato1@unime.it (E.T.); 2 Department of Agriculture, Food Science and Environment (Di3A), University of Catania, 95127 Catania, Italy; paolo.guarnaccia@unict.it; 3 Chromaleont s.r.l., c/o, Department of Chemical, Biological, Pharmaceutical and Environmental Sciences, University of Messina, 98168 Messina, Italy; 4 Unit of Food Science and Nutrition, Department of Medicine, University Campus Bio-Medico of Rome, 00128 Rome, Italy

**Keywords:** hempseed flour, hempseed oil, lipids, fatty acids, triacylglycerols, GC-FID/MS, UHPLC-MS, linear retention indices, industrial hemp

## Abstract

The growing demand in natural matrices that represent a source of dietary and nutraceutical molecules has led to an increasing interest in *Cannabis sativa*, considered to be a multipurpose, sustainable crop. Particularly, the considerable content in essential fatty acids (FAs) makes its derived-products useful food ingredients in the formulation of dietary supplements. In this research, the FA and triacylglycerol (TAG) composition of hempseed oils and flours were investigated using gas chromatography coupled to mass spectrometry and flame ionization detection as well as liquid chromatography coupled to mass spectrometry (LC-MS), respectively. Furthermore, a recently introduced linear retention index (LRI) approach in LC was successfully employed as a useful tool for the reliable identification of TAG species. A total of 30 FAs and 62 glycerolipids were positively identified in the investigated samples. Relative quantitative analyses confirmed linoleic acid as the most abundant component (50–55%). A favorable omega6/omega3 ratio was also measured in hemp-derived products, with the α-linolenic acid around 12–14%. Whereas, γ-linolenic acid was found to be higher than 1.70%. These results confirm the great value of *Cannabis sativa* as a source of valuable lipids, and the further improvement of the LRI system paves the way for the automatization of the identification process in LC.

## 1. Introduction

*Cannabis sativa* L. is one of the most ancient cultivated plants due to its extensive employment as a source of textile fiber (clothing, papermaking and sailmaking) and its use in folk medicine, to treat a wide range of ailments [1]. Currently, its popularity is mostly related to the “recreational” use, due to the high content of psychotropic substances. In fact, according to the World Health Organization, Cannabis is the most commonly cultivated, trafficked and abused illicit drug worldwide [2]. According to usage, *Cannabis sativa* plants are generally classified by drug-type and fiber type; in detail, the first category comprises the “non-medical, retail or recreational cannabis”, used for illicit purposes and the “medical cannabis”, employed for therapeutic scopes. 

Conversely, the second category comprises “industrial hemp”, which is used as starting material in food, cosmetic, construction, biocomposite and textile fields [3]. However, active substances responsible for psychotropic effects (e.g., Δ9-tetrahydrocannabinol, THC) are predominantly located in female flowers and resin-producing trichomes, which show the concentration of THC as higher at 20%. Dried stems usually are characterized by amounts of psychotropic substances lower than 0.3%; instead, roots and seeds do not contain THC [3]. Nevertheless, root- and seed-derived products can be viewed as valuable components because of their potential use as a source of bioactive molecules and raw material for industrial purposes.

The term hemp refers to *Cannabis sativa* cultivars grown for industrial purposes, characterized by levels of THC; <0.2% (EU Regulation 1307/2013) [4]. The EU Plant variety database of the European Commission contains the hemp varieties (>70) registered as ‘‘Agricultural Species-Varieties’’, which can be cultivated in EU; among them Futura 75, Uso 31 and Finola are the most used cultivars for industrial purposes [5].

Among the different parts of the plant that can be conveniently used for many industrial and economic purposes, hemp seeds have shown a favorable composition in ω-6 and ω-3 polyunsaturated fatty acids (PUFAs), especially linoleic acid and α- and γ-linolenic acids, high-quality and easily digestible proteins rich in essential amino acids (e.g., edestin and albumin), vitamins (e.g., tocopherols), antioxidant molecules (e.g., polyphenols) and minerals (e.g., potassium, magnesium and calcium) [6,7].

The considerable content in ω-6 and ω-3 PUFA represent a key factor in determining the great value of the plant; in fact, as is well known, these components are involved in many biological pathways and positively affect human health by contributing to the regulation of human metabolic activities and by preventing cardiovascular diseases [8]. Lastly, the favorable ω6/ω3 ratio, around 3:1, makes this product highly beneficial for human consumption [9]. Hence, hemp seeds and their derived products, such as oil and flour, may represent a promising source of high value molecules for the potential daily use as dietary supplements and for the production of nutraceuticals.

Several studies aimed at the determination of the lipid profile of *Cannabis sativa* products, concerning the total FA composition [10,11,12]. However, only a limited number of studies have been aimed at the characterization of complex lipids, such as triacylglycerols (TAGs) in this species [13]. Nevertheless, the study of complex lipids in their native form is proved to be crucial to obtain additional information on lipids role and on FA arrangement into each species.

Regarding lipid analysis, FAs are generally investigated, in form of FA methyl ester (FAME) derivatives, by gas chromatography (GC) coupled to flame ionization detection (FID) and mass spectrometry (MS), for quantitative and qualitative purposes, respectively [14,15]. Instead, reversed phase liquid chromatography (RP-LC) is the most suitable separation approach for complex lipid analysis, as in the case of TAGs [16,17,18,19]. In RP-LC, hydrophobic interactions between the target analytes and the stationary phase, normally a octadecylsilica (C18) particle packed column, provide a separation of the mixture that directly depends on the TAG partition number (PN), calculated from the equation PN = ECN = CN—2DB (equivalent carbon number, carbon chain length and double bonds number, respectively) [20].

The present work aimed at the evaluation of the lipid composition of different hemp-derived products (seed oil, seed flour and seed flour residue) by means of GC-FID/MS and LC-MS analytical methods. Moreover, a recently introduced linear retention index (LRI) approach in LC [19,21] was employed to achieve a reliable identification of TAGs in the examined samples. In this way, the identification process in LC was based on a dual filter approach that is quite similar to GC-MS methods. It exploits the complementarity between the information obtained from mass spectra and retention data in the form of LRI, which is a parameter as independent as possible from operating conditions so that it is reproducible at both intra- and inter-laboratory levels [21]. 

In order to achieve a fast and automatic peak assignment, an in-house spectral library with embedded LRI values was utilized and, according to the dual filter search, compounds falling out from the LRI window were automatically excluded from the MS search. This is of greater importance in LC than in GC, due to the poorly informative MS spectra normally obtained through atmospheric pressure ionization sources, which mainly provide the molecule-related ion information so that more isomers can be assigned to a single peak.

The use of this innovative tool can surely improve the knowledge about intact lipids in hemp-derived products, also making the analytical methods more user-friendly and broadly applicable from industries. Moreover, the findings about a favorable ω6/ω3 ratio and the high content of both ω6 and ω3 FAs, readily metabolizable as TAGs, confirmed the importance of these products for nutraceutical usage.

## 2. Results and Discussion

### 2.1. Fatty Acid Methly Ester Analysis

#### 2.1.1. Choice of the Chromatographic Method and Qualitative Results

The increasing interest in hempseed oil may be due to the impressive content in unsaturated FAs, which can be higher than 90%. Hemp-derived products are rich in linoleic acid (L, 18:2ω6) and α-linolenic acid (Ln, 18:3ω3, commonly abbreviated as ALA) defined as essential fatty acids (EFAs) [22]. Moreover, hempseed oil is reported to contain remarkable amounts of stearidonic acid (St, C18:4ω3, often abbreviated as SDA) [9,23] commonly found in marine organisms and γ-linolenic acid (γLn, C18:3ω6, often named GLA,) [24,25], which has been found in a few vegetable oils, including primrose, borage and blackcurrant oils [22].

As can be inferred, these two FAs are not widespread in conventional consumed edible oils. Such reports suggest for hemp a more complex FA profile with respect to common vegetable oils (soybean, corn, olive, peanut, palm and sunflower); the latter are characterized by linoleic, oleic and palmitic acids that account for approximately the 90% of the total FA composition. As a consequence, a proper chromatographic method is required to attain satisfactory peak resolution for single FAs.

In recent years, our research group successfully exploited the selectivity of ionic liquid (IL) stationary phase for GC analyses, reporting very reproducible results in terms of LRI values, thus, allowing for a reliable peak identification, even when changing the instrumental setup [15,26]. In addition, the dual-filter MS/LRI library enabled univocal peak assignment for all FAs, including the isomer pairs C18:1ω9/C18:1ω7 and C18:3ω3/C18:3ω6 [26].

**Figure 1:**
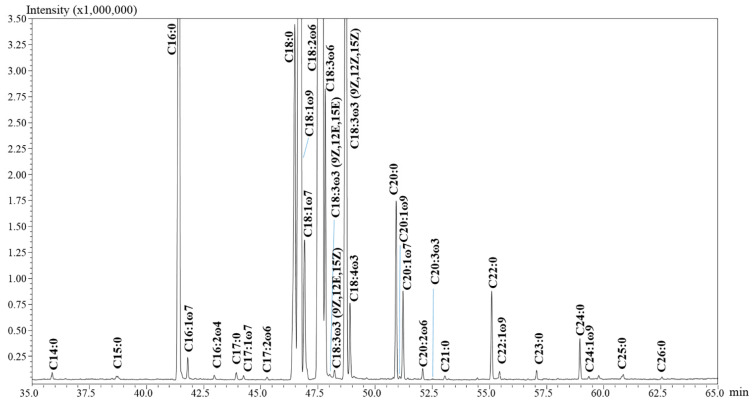
Thirty-minute expansion of the GC-FID chromatogram relative to the FA profile of a hempseed oil. FAs were detected as methyl esters (FAMEs).

In this research, the method previously developed on the IL-60 column by GC-MS/FID analyses was employed for the elucidation of the total FA profile in hempseed oil (*n* = 4), hempseed flour (*n* = 2) and flour residue (*n* = 1), after the conversion of lipids into less polar and more volatile FAMEs. Since all the investigated samples showed similar qualitative profiles, Figure 1 reports the GC-FID profile obtained for only one of them.

It should be also mentioned that the lipid extracts of hempseed products also contain minor lipid compounds, such as sterols and tocopherols, which do not undergo any derivatization reaction, which is a transesterification that only involves the saponifiable fraction (i.e., glycerolipids and phospholipids). On the other hand, unsaponifiable compounds, which are not eluted from the IL column under the employed GC conditions, were neglected in the present study and will be the object of a future work.

As for flours and flour residue, the extraction yield was around 1–2%, since 500–900 mg of oil were obtained from 40 g of solid product.

Table 1 shows the identified FAs in all the samples, along with the experimental and tabulated LRI, the mass spectral similarity and the relative quantification. A total of 30 FAs were positively identified with a spectral similarity higher than 90% for all the compounds and a difference between experimental and tabulated LRI of less than 5 for most of the compounds, including three isomers of the ω3 linolenic acid, which differ for the E/Z configuration at one or two double bonds. The possibility to separate cis/trans isomers is one of the key features of IL columns, which, already in previous works [27,28,29,30], allowed to discriminate all eight isomers of linolenic acid by using longer 100 or 200 m columns.

#### 2.1.2. Quantitative Results

From a quantitative point of view, linoleic acid was the most represented FA in hemp-derived products, followed by oleic and α-linolenic acids. In detail, linoleic acid ranged from a minimum of ~53–53.5% in one of the analyzed oil (producer IV) and in the residue of the flour production provided by the same company to a maximum of ~56.5% in another oil; similarly, the minimum amount of α-linolenic acid was registered in the flour residue (11.56%), followed by the oil (12.13%) produced by the same company (producer IV), while the maximum amount was detected in oil I (13.85%); conversely, oleic acid was quantified at the minimum level in oil I (13.86%), reaching a significantly higher content (*p* < 0.01, by running an ANOVA) in oil IV and the corresponding flour residue (average values of 18.31% and 18.40%, respectively).

According to these results about major FAs, it is possible to pinpoint that the lipid fraction of hemp-derived products is mainly composed by unsaturated FAs, which account for nearly 90% in all the investigated samples, with PUFAs as more than triple the monounsaturated FAs (MUFAs), as reported in Table 1. In addition, a ω6/ω3 ratio around 4:1 was calculated in all the samples, with minimum average values of 4.0 in oils I and III and maximum average values of 4.6 in the residue provided by producer IV. Despite C18:2ω6 and C18:3ω3 comprising most of the PUFA content, the contributions of other ω6 and ω3 FAs need to be considered for the comprehensive evaluation of the beneficial properties of hempseed-derived products. 

Among the ω6 series, significant levels of γ-linolenic acid (GLA) were found, going from an average percentage of 1.49% for oil II to 2.58 for oil I, passing through average values around 2% for the other samples. The use of GLA as dietary supplementary has become more frequently in the last decades due to the evidence of the anti-inflammatory effect exerted by this ω6 FA [31]. In fact, it is rapidly converted by the action of an elongase enzyme into the ω6 eicosatrienoic acid (C20:3ω6), known as dihomo-γ-linolenic acid (DGLA), which, in some way, act as the ω3 FAs, being precursor of prostaglandins with anti-inflammatory activity [31]. In the human body, DGLA inhibit the biosynthesis and contrast the effect of the eicosanoids generated through the pathway of the ω6 FAs, which are pro-inflammatory molecules [31].

As for the ω3 series, stearidonic acid represented the second most representative compound, with a mean value of 0.4% in oils II and IV up to 0.75 for oil I. The latter resulted in the most concentrated sample in ω3 FAs, leading to the most beneficial ω6/ω3 ratio, while the minimum amount of these FAs in the lipid fraction of the flour residue made it the less convenient from this point of view. Such a difference in the ω3 levels is offset by an opposite trend in the content of oleic acid, which accounts for nearly the totality of monounsaturated FAs (MUFAs). 

They were present at significantly higher percentages in the flour residue and in oil IV (*p* < 0.01, by running an ANOVA) and at a lower percentage in the oil I (~5% of difference between oil I and the flour residue). The residue and oil IV were less concentrated in PUFAs (mean values of 67.63 and 68.07, respectively), which correspond to the sum of ω6 and ω3 FAs, while oil I had the maximum levels of 73.49% ± 0.11%, being the most concentrated in both ω6 and ω3 FAs.

Finally, the low content of saturated FAs (SFAs) also makes all the food products under investigation extremely interesting from a nutraceutical point of view. As can be observed in Table 1, saturated FA (SFA) were found to be poorly present in hemp-derived products, with a percentage content lower than 12.5% for all the samples; with maximum levels in the flour residue (mean value 12.30%) and minimum amount in oil II (mean value 10.51%).

Despite the observed differences between the analyzed samples, quantitative data clearly point out the beneficial properties of hempseed products, including oilseed, flours and corresponding residues, all characterized by low percentages of SFAs, very high percentages of PUFAs with a favorable ω6/ω3 ratio. The moderate percentage of MUFAs, higher than SFAs, also gives a positive contribution to the well-being, due to their potential in reducing the risk of cardiovascular disease [32]. 

In this regard, the SFA/MUFA ratio, below 0.75 for all the samples, further confirms the beneficial effect of hempseed-derived products, despite it being higher with respect to the most common vegetable oils (olive oil, soybean, sunflower, etc.) due to minor amounts of oleic acid [33]. On the other hand, a PUFA/SFA ratio ranging between 5.4 and 6.8 was calculated for all the samples, which is higher than the above-mentioned edible oils, due to the higher amount of ω3 PUFA and/or lower content of SFAs. 

In order to consider the combination of both ratios and assess some functional properties of the investigated food products, three nutritional indices, i.e., atherogenic index (AI), thrombogenic index (TI) and the ratio of hypocholesterolemic and hypercholesterolemic FAs (h/H), were calculated (Table 1). The first one represents the ratio between the sum of some saturated FAs and unsaturated FAs (both MUFA and PUFA), removing stearic acid (C18:0) from SFA since it is not considered an atherogenic acid according to experimental evidence [34]. 

Values close to zero are highly desirable to prevent the risk of atherosclerosis. In this study, AI was 0.07–0.08 in all the cases, which resulted as smaller compared to the value reported in literature for olive oil (AI = 0.14), marine organisms and seaweeds (high variability depending on the species), while a comparable value was reported for sunflower oil (AI = 0.07) [34,35]. 

As for TI, compared to the simplest SFA/PUFA ratio, we considered different multiplying factors for each unsaturated FA class so that the ω3 species provided the highest contribution. Even in this case, values close to zero indicate a low risk of thrombus formation. In this study, TI ranged between 0.11 and 0.14, which is smaller than the values obtained for more common vegetable oils (both olive and sunflower oil, TI = 0.32 and 0.28, respectively) [34] and marine organisms (TI = 0.16 for mackerel and up to 0.74 for tuna) [34,36] but higher than linseed oil (TI = 0.07), which was characterized by alpha-linolenic acid as the most abundant FA [37]. Finally, the h/H ratio was calculated to estimate the hypocholesterolemic effect of hempseed products. 

The higher the value obtained, the greater the effect. In this case, significant differences existed between the investigated samples (*p* < 0.01, by running an ANOVA). In particular, oil II had the highest ratio, mainly due to the lowest level of palmitic acid, while oil IV and the flour residue showed the smallest h/H ratio because of highest palmitic acid amount and lowest PUFA content. However, the obtained values, in the range of 11.5–14 were significantly higher than for the marine organisms reported in recent works [35,38] and comparable to linseed oil (h/H = 14.75) [37]. 

**Figure 2:**
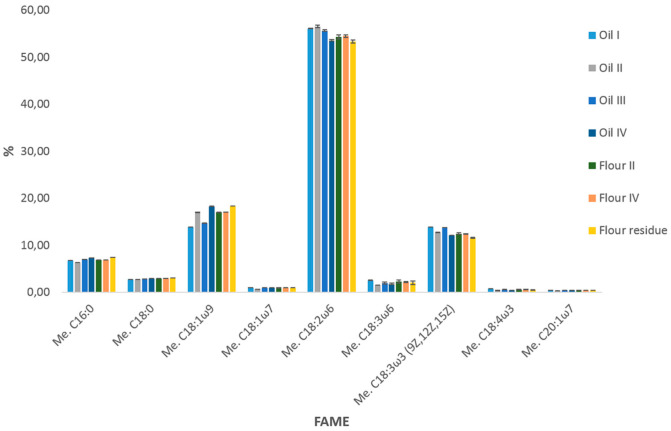
Comparative histogram (% content) of major FAs in the lipid fraction of hempseed products.

In order to better visualize the quali-quantitative composition of the analyzed samples and highlight other differences, Figure 2 reports the comparative histogram relative to the content of the main FAs (≥0.4%). It can be immediately pinpointed that the two flours (producers II and IV) have quite identical lipid compositions (*p* > 0.01, by running an ANOVA), while some differences were observed with respect to the corresponding oils and the lipid extract of the by-product. 

In particular, lower levels of palmitic and oleic acids were detected in flour IV compared to both the oil and the residue (*p* < 0.01, by running an ANOVA), offset by a higher level of all the ω6 and ω3 FAs (*p* < 0.01, by running an ANOVA), thus, justifying the higher total PUFA, ω6 and ω3 amounts reported in Table 1. Conversely, flour II resulted as less concentrated for the corresponding oil in linoleic acid (*p* < 0.01, by running a two-tailed *t*-test) and more concentrated in γ-linolenic and stereadonic acid (*p* < 0.05, by running an ANOVA). Saturated FAs were more represented in the flour (*p* < 0.01, by running a two-tailed *t*-test).

Considering that different hemp seeds generated flours of very similar FA composition but oils with different FA profiles, it is possible to conclude that the detailed elucidation of the lipid composition of hempseed-derived products can be extremely useful for hemp companies to understand how the sample treatment and production technologies can affect the nutritional profile of the final product.

### 2.2. Triacylglycerol Analysis

#### 2.2.1. Choice of the Chromatographic Method

Among lipid components, TAGs represent the most abundant constituent of plant seed oils and derived food products [39]. The separation of such components, which derive from the quite randomized combination of FAs bound to the glycerol skeleton, can represent a challenging analytical task, especially when dealing with complex mixtures in which numerous species show the same PN values and thus similar chromatographic behavior. In this respect, as discussed in the previous section, hempseed products resulted more complex matrices compared to most common edible oils obtained from vegetable sources. 

Consequently, analytical methods providing high chromatographic efficiency and resolution are highly demanded. The TAG analysis of hempseed oil by LC-MS has been poorly described in scientific literature [13,40,41,42]. In most of the few cases reported, the analytical workflows were based on a preliminary LC separation, which allowed for the collection of the most abundant fractions, each corresponding to a single PN; then, the fractions were injected into a MS system for TAG identification and/or subjected to transesterification or enzymatic procedures to obtain the FA composition of each fraction. In this way, only the most abundant species for each PN were identified [40,41,42]. 

Moreover, these procedures are time consuming and difficult to automatize. To the best of the authors’ knowledge, the research conducted by Lisa et al. is the only work reporting the detailed elucidation of intact TAGs from hempseed oils by non-aqueous reversed-phase liquid chromatography (NARP-LC) online coupled to MS via an APCI (atmospheric pressure chemical ionization) ion source. 

In the reported application, the high-resolution chromatographic separations were performed on two serially coupled C18 columns (total length 45 cm by coupling a 30 cm and a 15 cm, both 3.9 mm ID) and acetonitrile/2-propanol were employed as mobile phases at a flow rate of 1.0 mL/min [13]. In this research, TAG NARP-LC separation was achieved by serially coupling two C18 (total length 20 cm) narrow-bore columns, allowing the use of reduced operational flow rates (0.5 mL/min). The employed analytical conditions were previously optimized [19], thereby, affording a satisfactory compromise between the analysis time and chromatographic efficiency.

Even in this case, minor lipid components, such as sterols, tocopherols and phospholipid, were not detected, due to the method sensitivity and/or to the use of NARP conditions, which are more specific for the elution of non-polar compounds.

In addition, the recently implemented LRI approach was applied, aiming to simplify the identification procedure, thus, integrating in a single and automated platform the main analytical operations: chromatographic separation, MS detection, peak identification and integration.

Therefore, the TAG composition of hempseed products (four oils, two flours and one flour residue) was exhaustively investigated for the first time in a reasonable analysis time and through a user-friendly and automatic identification strategy, which employed a dual-filter algorithm by simultaneous search into an LRI database and an MS library.

**Figure 3:**
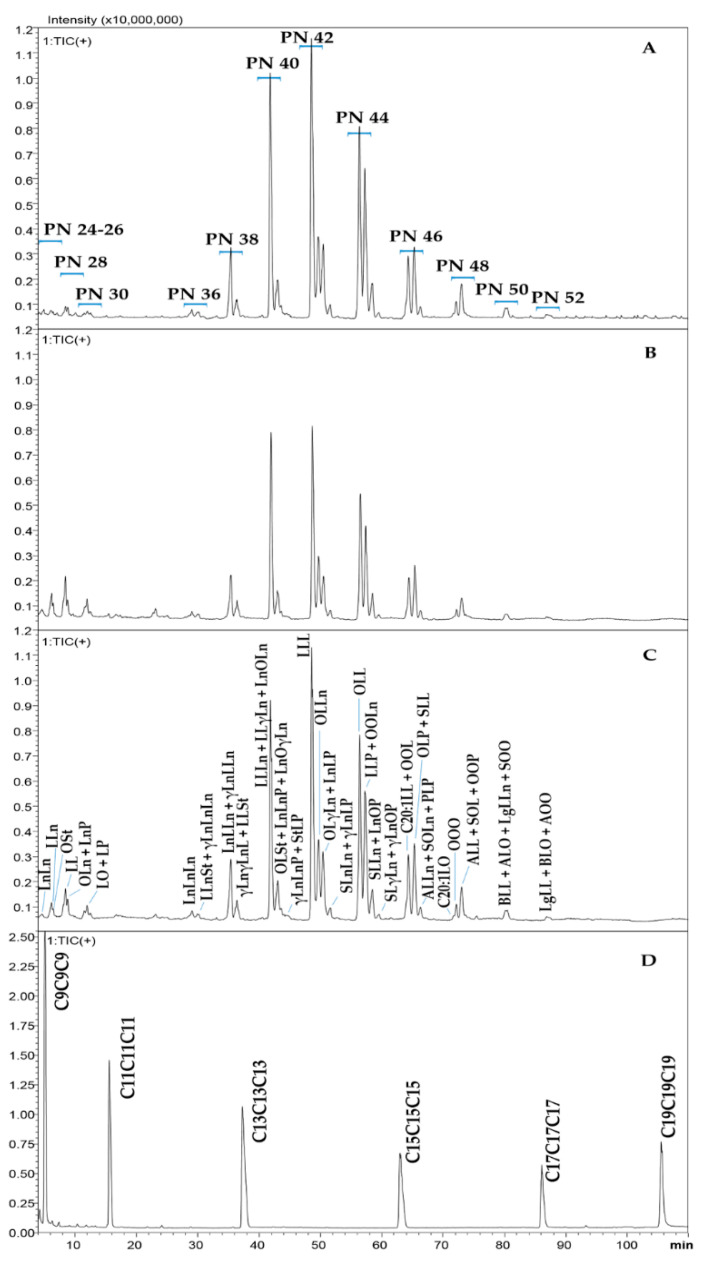
LC-MS profiles of (**A**) hempseed oil IV, (**B**) hempseed flour IV, (**C**) flour residue IV and (**D**) reference homologue series. PN is the partition number of lipid species. Fatty acid abbreviations: C9: nonanoic acid; C11: undecanoic acid; C13: tridecanoic acid; C15: pentadecanoic acid; C17: heptadecanoic acid; C19: nonadecanoic acid; Ln: linolenic acid (C18:3);L: linoleic acid (C18:2); O: oleic acid (C18:1); St: stearidonic acid (C18:4); P: palmitic acid (C16:0); S: stearic acid (C18:0); A: arachidic acid (C20:0); B: behenic acid (C22:0); and Lg: lignoceric acid (C24:0).

As in the case of the GC-FID/MS results, the TAG chromatograms of all the investigated products showed negligible differences by visual inspection. Then, only the profiles of the oil, the flour and the residue provided by the same company are reported in Figure 3A–C. From an analytical point of view, a satisfactory chromatographic resolution between different PN regions and within the same PN value was achieved. More in detail, PN ranged from 36 to 52 for TAGs, while minor peaks of diacylglycerols (DAGs) were identified in the PN regions from 24 to 30, mostly expressed in the flour (Figure 3B) and in the residue (Figure 3C), here investigated for the first time. Indeed, DAGs were not reported in previous works dealing with hempseed oils. The chromatogram of the reference homologue series used for LRI calculation is shown in Figure 3D to highlight the proper coverage of the entire chromatographic space. 

Each PN region can be easily assigned on the basis of the comparison between the retention time of the reference standard compounds and those of the unknown peaks in the samples. Particularly, each odd carbon chain number reference compound is eluted few seconds earlier or later than the subsequent or previous even carbon chain number TAG. Moreover, it can be highlighted that even PN regions, as well as the reference TAGs are quite equidistant from each other under the linear gradient used for the LC separation, thus, guaranteeing the proper functioning of the LRI strategy [21].

Considering that, in RP-LC, TAGs are eluted in the order of ascending PN values, the first 50 min of the chromatogram includes species containing mainly PUFA, with a total DB number ≥ 5 (Table 2), with the exception of few DAGs; specifically, the ω3 FAs ALA and SDA and the ω6 FAs GLA are highly present in the PN regions 24–42. On the other side, the last eluted compounds are TAGs containing mainly SFAs and MUFAs; particularly, the PN regions 46–52 contain longer carbon chain FAs, such as arachidic (C20:0), gondoic (C20:1n9), behenic (C22:0) and lignoceric acid (C24:0). However, they represent minor peaks compared to the most representative TAGs in the PN regions 38–44.

#### 2.2.2. Qualitative Analysis: Application of a Novel LRI-Based Identification Strategy

The identification was primarily performed in an automatic way by using the novel ChromLinear software, which enables a fast data processing by matching with a previously built and constantly implemented LRI database [18,19,43] and home-made MS library reported in a previous work [43].

It is noteworthy that the Δ value between the experimental and tabulated LRI values (LRI_exp_ and LRI_tab_, respectively) for almost all the compounds remained inside the tolerance window of ± 15 units, as derived from previous calculation about the separation number of the applied chromatographic method [19,21]. Out of a total of 62 identified compounds, 31 TAGs were positively matched with the LRI database (ΔLRI ≤ 15), and an additional 4 TAGs differed from the tabulated values of 17–20 units, mainly due to coelutions or very low peak intensity.

The LRI parameter resulted in being very helpful in the discrimination of TAGs containing different isomers of linolenic acid, Ln or γLn, characterized by identical MS spectra. Specifically the TAG LnLnLn (LRI_exp_ 3675) was unambiguously identified since the isomer γLnγLnγLn is tabulated with a totally different LRI value (3747); similarly the isomeric pairs LnLLn and γLnLγLn, OLLn and OLγLn, LnLP and γLnLP, LnOP and γLnOP were discriminated because of different LRI values, despite the difference in LRI_tab_ decreases with increasing PN, viz. retention factor under RP conditions.

Regarding mixed TAGs containing both isomers, species containing exclusively αLn or alternatively γLn were present in our previous database, while the investigated samples resulted as within reach in TAG containing both the α and γ isomers, as reported by Lisa et al. [13] and in accordance with the GC profile discussed in the previous section. 

In such cases, the experimental LRI showed intermediate values between the TAGs containing only one of the isomers. Specifically, it is already known that TAGs containing γLn show higher retention than in the TAGs containing the αLn isomer counterpart, due to a slightly higher hydrophobicity of this configuration of double bonds [13,44]. The awareness of such behavior allowed the reliable identification of TAGs containing both Ln and γLn as in the case of the isomers γLnLnLn and γLnγLnLn, characterized by identical MS spectra, even compared to LnLnLn. Within this context, the analyses of hempseed products contributed to the previous database, with these compounds present in more samples and also confirmed by the literature [13]. 

According to the GC profile (Ln is around six times more abundant than γLn), peaks identified as TAGs containing Ln were more intense than those relative to the elution of the isomers containing γLn, as well as the isomer in which Ln is entirely replaced by γLn were poorly identified, while more often only one position of the glycerol backbone was esterified with γLn (e.g., LnOγLn and γLnLnP, while γLnOγLn and γLnγLnP were not identified). Finally, γLn-containing TAGs were not detected at PN ≥ 46, in which Ln-containing TAGs were scarcely represented.

Compounds univocally identified on the basis of the LRI criterion are marked with * in Table 2. However, they were also confirmed through MS search. In the case of ambiguous identification based on LRI values, the MS library drove the selection of the right candidate. Afterward, since both databases are still not exhaustive, a manual interpretation of MS spectra was also conducted to detect species not included in the libraries. 

The manual identification of acylglycerols in LC-MS is quite feasible through the commonly employed APCI source, which provides structural information deriving from TAG protonated species [M + H]^+^ and fragment ions originated from the loss of the fatty acyl groups esterified to the glycerol moiety [45]. In addition, it is necessary in the case of coelutions to confirm the presence of more compounds falling within the same LRI window.

**Figure 4:**
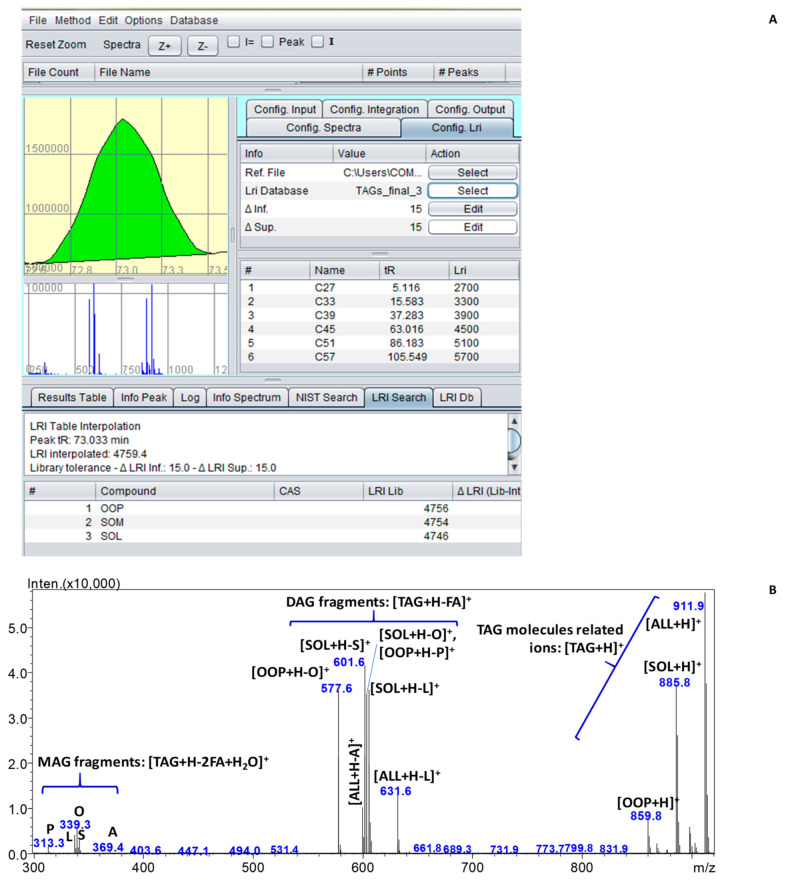
(**A**) Automatic search in the LRI database for peak at 73.03 min and (**B**) relative MS spectra with the assignment of MS signals.

For the sake of clarity, Figure 4A reports the automatic search into the LRI database by the ChromLinear software for peak at 73.03 min. Only three candidates are listed, namely dioleyl-palmitin (OOP), stearyl-oleyl-myristin (SOM) and stearyl-oleyl-linoleyl (SOL). The second one could be reasonably ruled out according to the GC profile, which showed only trace amount of myristic acid (M, C14:0). However, the interpretation of the mass spectrum in Figure 4B definitely excluded SOM and confirmed the coelution between SOL and OOP. 

In addition, other MS signals were detected and assigned to the molecule-related ion of dilinoleyl-arachidin (ALL) and their diacylglycerol (DAG) and monoacylglycerol (MAG) fragments. Despite that the MS similarity with the spectral library cannot be reliable because of the coelution issue, the TAG SOL, which provided higher MS signals compared to OOP, was identified with almost 50% probability.

It is noteworthy that the restricted list of candidates generated by the LRI search is helpful for the subsequent step of interpretation of the MS spectrum by driving the analyst in the correct assignment of each fragment.

Figure 4A also shows a screenshot of the novel software, which enables both qualitative and quantitative analyses through the complementarity of LRI and spectral data and peak integration, respectively. The input files (chromatogram of the reference homologue series) for the automatic LRI calculation and search are uploaded in the dedicated right panel of the software so that a table containing the retention time of reference compounds is automatically filled with z numbers (i.e., the PN of each reference compound, from 27 for trinonanoin C9C9C9 to 57 for trinonadecanoin C19C19C19) and the LRI arbitrarily assigned (i.e., z * 100).

#### 2.2.3. Quantitative Results

Along with the qualitative information about the identified compounds, Table 2 shows the percentage content of each species.

As it is well known, the relative response of TAGs by APCI-MS depends on the number and positions of double bonds and on the lengths of acyl chains of FAs, which are arranged in the formation of each TAG. Therefore, the relative abundances of the investigated compounds were corrected by the employment of response factors (RFs) aiming to minimize the differences in terms of the ionization efficiency. In detail, the RFs of TAGs referred to the RF of triolein (OOO), considered as equal to 1.00, according to the procedure proposed by Holčapek et al. [17].

The TAG profile for both hempseed oils and hemp flour products resulted as in agreement with the FAMEs profile obtained by GC-FID/MS. As an example, the most abundant FA in all the analyzed samples was linoleic acid (L, C18:2n6), and the high amount of this component resulted in the predominant presence of L containing TAGs, with LLL as the most represented species in the lipid fraction of hempseed-derived products with not significant differences (*p* > 0.05, by running an ANOVA) between hempseed oils and flours. 

Specifically, LLL is comprised between mean values of 19.35% and 20.31% in the first case and ranging from 17.12% to 18.31% for the latter, passing through an intermediate value of 19.18% in the flour residue. A similar trend, even if not identical, was observed for other L-containing TAGs, such as LLLn, LLγLn, LnLLn and γLnLLn, showing maximum amounts for oils I, II and III and minor concentrations for the other samples. This behavior reflects the differences in the percentages of L quantified by GC (as in Figure 2). The flour residue was the less concentrated in Ln-containing TAGs, in most cases followed by oil IV, the flours and oil II. 

Remarkably, these samples were characterized by a higher ω6/ω3 ratio (as from GC results in Table 1) compared to oil I and III. In this regard, LC analyses confirmed that the latter were the richest samples in St-containing TAGs, such as LLnSt and LLSt. Mixed TAGs containing oleic and linoleic, such as OLL and OOL, showed the trend observed by GC for oleic acid with minimum amounts for oil I and the highest percentages for oil IV and the flour residue.

TAGs containing only saturated FAs were not detected in any samples since they are always combined with mono- (O) or polyunsaturated FAs (L and Ln), even in the later eluted compounds, falling into the PN regions between 48 and 54, accounting for around 4% of the total lipid composition. This finding, arising from the analysis of lipids in their native form as they are absorbed and metabolized from living organisms, confirms the beneficial roles of these food products for human health.

**Figure 5:**
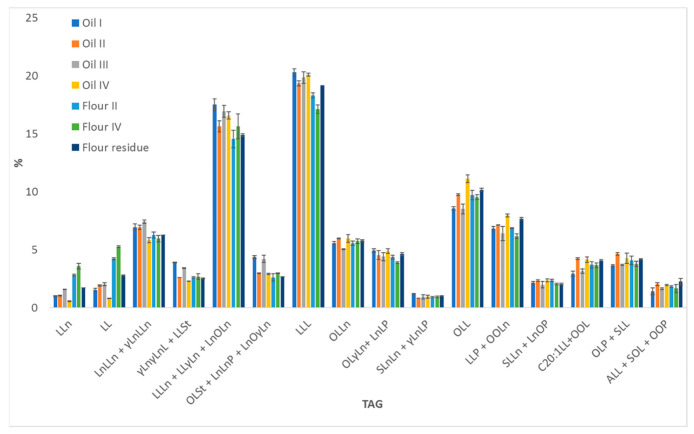
Comparative histogram (% content) of major DAGs and TAGs in the lipid fraction of hempseed products.

For an immediate visualization of the quantitative results, the histogram reporting the comparison of the relative content of the main DAGs (≥0.5%) and TAGs (≥1.0%) between the investigated samples is depicted in Figure 5. Flours are richer in DAGs compared to oils, likely due to a partial hydrolysis occurring in the seeds after the oil extraction. As for TAGs, oils I and III showed the most similar composition (*p* > 0.05 by running an ANOVA). 

Oils II and IV provided also quite similar quantitative profiles, even comparable in the content of some TAGs to the lipid extracts of both flours and the residue, which derived from the same seeds. Conversely from GC analyses, which did not reveal significant differences between the two flours, LC analyses highlighted a major content of some TAGs in flour II, while flour IV was richer in the corresponding DAGs. 

Such results could be especially meaningful for the evaluation of the storage conditions, since bad sample preservation could promote the hydrolysis of TAGs and the release of free FAs, which are more susceptible to oxidation, thus, leading to deterioration. From this point of view, the analysis of intact lipids can be more useful to monitor possible alteration of the lipid composition due to inappropriate sample treatments, occurring both at industrial levels and for quality control analysis.

In conclusion, the TAG composition represents an effective fingerprint that is unique for each lipid matrix due to the highest combinations of FAs that are arranged in glycerolipids. For this reason, this kind of analysis could be used in the near future to discriminate between a major number of hempseed-derived products, obtained by different companies and/or through different technologies and/or from different hemp varieties.

## 3. Materials and Methods

### 3.1. Reagents and Materials

Acetonitrile and 2-propanol (LiChrosolv^®^, hypergrade for LC−MS grade), *n*-heptane, *n*-hexane, methanol and potassium hydroxide (KOH) (reagent grade) were purchased from Merck Life Science (Darmstadt, Germany). Trinonanoin (C9C9C9), triundecanoin, (C11C11C11), tritridecanoin (C13C13C13), tripentadecanoin (C15C15C15), triheptadecanoin (C17C17C17) and trinonadecanoin (C19C19C19) standards and a 1000 μg/mL C4-C24 Even Carbon Saturated FAMEs mixture in *n*-hexane were also purchased from Merck Life Science (Darmstadt, Germany).

### 3.2. Samples

Hempseed oils were provided by Canapar (Ragusa, Italy), Canapuglia (Bari, Italy), Molino Crisafulli (Caltagirone, Italy) and Marishanti (Ragusa, Italy) companies, hempseed flours were from Canapar and Crisafulli, and hempseed flour residue was obtained by Canapar. Specifically, oil samples were obtained by cold pressing hemp seeds (without any filtration); flour samples were obtained by the stone milling of hemp seeds after cold pressing and oil extraction; and the residue from the processing of the flour was obtained by sieving the raw product after oil extraction.

### 3.3. Sample Preparation

Hempseed oils were diluted in 2- propanol (1000 mg/L) for LC-MS analysis. Seed flours and seed flour residue lipids were extracted by solvent maceration. Briefly, 50 mL of *n*-hexane were added to 40 g of flour and residue, and the mixture was allowed to stand for 40 min. The hexane phase was collected and evaporated by rotary evaporator to obtain the lipid extract; then, a 1000 mg/L solution was prepared by dissolving 10 mg of oil or lipid extract in 10 mL of 2-propanol, used as the sample diluent for LC-MS analysis.

For GC-MS/FID analysis, all samples were subjected to a cold derivatization procedure by using KOH in methanol (2M) to convert intact lipids into the corresponding fatty acid methyl esters (FAMEs). Briefly, 200 µL of KOH in methanol (2M) and 2 mL of *n*-heptane were added to 100 mg of oil into a 4 mL screw vial. The mixture was vortexed for 30 s. Then, the *n*-heptane upper layer was collected into a 2 mL vial for GC-MS/FID analysis.

### 3.4. Instruments and Analytical Conditions

#### 3.4.1. GC-MS/FID

GC-MS analyses were performed on a GCMS-QP2020 instrument (Shimadzu, Duisburg, Germany) equipped with a split-splitless injector (280 °C) and an AOC-20i autosampler. Separations were performed on a SLB-IL60 30 m × 0.25 mm id, 0.20 μm *d.f.* capillary column (Merck Life Science), and the temperature program was set as follows: 50 to 280 °C at 3.0 °C/min. The injection volume was 0.5 µL with a split ratio of 1:50. Helium was employed as carrier gas, with an average linear velocity of 30 cm s^−1^ and an initial inlet pressure of 26.6 kPa.

The following MS parameters were employed: mass range, 40–550 amu; ion source temperature, 220 °C; interface temperature, 250 °C; and event time, 0.20 s. Data were acquired and processed by using GCMSsolution ver. 4.50 software (Shimadzu Europa, Duisburg, Germany). Specifically, the identification procedure was performed automatically by the software, based on a dual-filter LRI/MS search algorithm after the loading of the LIPIDS Mass Spectral Library ver. 1.0 (Shimadzu Europa, Duisburg, Germany) in which LRI values calculated on the SLB-IL60 column after the injection of pure standards and the C4–C24 standard mixture as reference homologue series were embedded. Peak assignment was based on a MS spectral similarity higher than 85% and a ±10 LRI tolerance window.

GC-FID analyses were conducted on a GC-2010 instrument (Shimadzu, Duisburg, Germany) equipped with a split-splitless injector, an AOC-20i/s autosampler and an FID detector. The analytical conditions in terms of GC column, temperature program, carrier gas linear velocity and volume injection were the same as for the GC-MS analyses. The initial inlet pressure was 99.4 KPa. The FID parameters were: temperature, 280 °C; sampling rate, 40 ms; gas flow rates of 40, 30 and 400 mL min^−1^ for H_2,_ make-up gas (N_2_) and air, respectively. Data were acquired and processed using the LabSolution ver. 5.92 software (Shimadzu, Duisburg, Germany). Analyses were performed in triplicate, and quantitative results are expressed as the average of the area percentages.

The atherogenic (AI) and thrombogenic (TI) nutritional indices and hypocholaesterolemic/hypercholaesterolemic ratio (H/H) were calculated according the equations proposed by Ulbricht and Southgate [34] and Santos-Silva et al. [46], respectively. The indices were calculated as reported below:

AI = [C12:0 + (4 × C14:0) + C16:0]/(ƩUFA), (1)

TI = (C14:0 + C16:0 + C18:0)/[(0.5 × ƩMUFA) + (0.5 × Ʃn6-PUFA) + (3 × Ʃn3-PUFA) + + (Ʃn3-PUFA/Ʃn6-PUFA)],(2)

h/H = (C18:1n9 + ƩPUFA)/(C14:0 + C16:0) (3)

#### 3.4.2. LC-MS

Analyses were performed on a Nexera UHPLC system coupled to an LCMS-2020 spectrometer through an APCI ionization source (Shimadzu Europa, Duisburg, Germany). The chromatographic system consisted of a CBM-20A controller, two LC-30AD dual-plunger parallel-flow pumps, a DGU-20A5R degasser, a CTO-20AC column oven and a SIL-30AC autosampler.

Separations were performed on two serially coupled Ascentis Express C18 10 cm × 2.1 mm, 2.7 μm *d.p.* columns (Merck Life Science, Darmstadt, Germany). The employed mobile phases were acetonitrile (A) and 2-propanol (B), and the linear gradient program was: 0 min, 0% B for 105 min, and 50% B, held for 5 min. The flow rate was 0.5 mL/min, the oven temperature was 35 °C, the sample diluent was 2-propanol, and the injection volume was 10 μL.

The following MS parameters, through APCI source in positive (+) ionization mode, were employed: interface temperature, 450 °C; DL temperature, 250 °C; heat block temperature, 300 °C; nebulizing gas flow (N_2_), 1.5 L/min; drying gas, 5 L/min; acquisition range, 250–1200 *m/z*(+). Data were acquired by using LabSolution ver. 5.95 software (Shimadzu Europa, Duisburg, Germany).

#### 3.4.3. LC-MS Data Processing

Since commercial software for LC-MS analyses do not allow the LRI calculation, LC-MS data were processed by the novel ChromLinear software (Chromaleont, Messina, Italy), which was developed in-house for the LRI handling in LC [18,21]. This allows to integrate and automatically calculate the LRI values of the analytes according to equation 4, employing the mixture of odd carbon number TAGs as references homologue series previously integrated and loaded in the dedicated window of the software [18,21]. To this regard, a 1000 μg/mL standard mixture of the odd carbon number triacylglycerols (from C9C9C9 to C19C19C19) in 2-propanol was injected daily.

(4) $$LRI=100\left[z+6\frac{t_{Ri}-t_{Rz}}{t_{R\left(z+6\right)}-t_{Rz}}\right]$$

*z* = PN of the reference TAG eluted immediately before the analyte (it ranges from 27 for C9C9C9 to 57 for C19C19C19); *t_Ri_*, *t_Rz_* and *t*_*R*(*z* + 6)_ are the retention times of the analyte and the reference TAGs eluted immediately before and after the analytes, respectively.

Then, a dual-filter identification strategy was applied by using a home-made MS spectral library and an LRI database that was previously built according to a process as similar as possible to the well-established approach used in GC-MS. An LRI tolerance of ±15 units was employed, according to the LRI variability, peak resolution and separation capability assessed in the previous work [19].

## 4. Conclusions

In this research, seven different hempseed-derived products were investigated by using GC-MS/FID and LC-MS analytical techniques, and a total of 30 FAs and 62 glycerolipids were identified, respectively. As a result, the favorable composition in the lipid components of *Cannabis sativa*, especially in terms of ω6/ω3 FAs, was confirmed, revealing the great economic interest of this species, including for nutritional and nutraceutical purposes. The remarkable content in FAs that are poorly represented in nature, such as GLA and SDA, contributes to the great value of these products, differentiating them from traditionally consumed edible oils.

Moreover, it has been possible to further increase the knowledge on the FA distribution in complex lipids, providing additional information on their native form, both in hempseed oils and hempseed flours. The lipid composition, especially the TAG profile, could be relevant to differentiate oil and flour samples and similar samples obtained by different companies.

As far as the LC-MS analytical workflow is concerned, the already existing LRI database was further implemented, and the LRI approach was successfully confirmed to be a powerful strategy to obtain a reliable identification of TAGs in combination to MS. This strategy was capable of reducing the number of possible candidates for compounds showing the same molecule-related ions by APCI-MS.

## Figures and Tables

**Table 1:** Identified FAMEs in the lipid fraction of hempseed-derived products, along with their MS similarity (%), comparison between experimental (exp) and tabulated (tab) LRI values and the relative content (%).

n.	Compound	LRI_exp_	LRI_lib_	MS % Similarity	Oil I	Oil II	Oil III	Oil IV	Flour II	Flour IV	Residue IV
**1**	Me. C14:0	1401	1400	96	0.03 ± 0.00	0.04 ± 0.01	0.04 ± 0.00	0.04 ± 0.00	0.05 ± 0.00	0.05 ± 0.01	0.05 ± 0.00
**2**	Me. C15:0	1503	1500	95	0.02 ± 0.00	0.02 ± 0.01	0.03 ± 0.00	0.03 ± 0.00	0.03 ± 0.01	0.04 ± 0.00	0.04 ± 0.01
**3**	Me. C16:0	1603	1600	97	6.76 ± 0.01	6.36 ± 0.03	7.01 ± 0.01	7.31 ± 0.00	6.94 ± 0.01	6.87 ± 0.00	7.46 ± 0.01
**4**	Me. C16:1n7	1618	1609	97	0.11 ± 0.00	0.03 ± 0.00	0.11 ± 0.00	0.12 ± 0.00	0.11 ± 0.00	0.11 ± 0.00	0.12 ± 0.00
**5**	Me. C16:2n4 (9Z.12Z)	1665	1663	90	0.02 ± 0.00	0.09 ± 0.00	0.02 ± 0.00	0.02 ± 0.00	0.01 ± 0.00	0.02 ± 0.01	0.02 ± 0.00
**6**	Me. C17:0	1703	1700	95	0.04 ± 0.00	0.05 ± 0.01	0.04 ± 0.00	0.04 ± 0.00	0.04 ± 0.00	0.05 ± 0.00	0.05 ± 0.00
**7**	Me. C17:1n7	1716	1713	94	0.02± 0.00	0.03 ± 0.00	0.03 ± 0.00	0.02 ± 0.00	0.03 ± 0.01	0.03 ± 0.00	0.03 ± 0.00
**8**	Me. C17:2n6 (9Z.12Z) ^a^	1757	-	-	0.03± 0.00	0.02 ± 0.00	0.04 ± 0.00	0.02 ± 0.00	0.02 ± 0.00	0.02 ± 0.00	0.03 ± 0.00
**9**	Me. C18:0	1806	1800	97	2.77 ± 0.01	2.78 ± 0.01	2.83 ± 0.01	2.97 ± 0.00	2.96 ± 0.01	2.97 ± 0.01	3.04 ± 0.01
**10**	Me. C18:1n9	1817	1810	94	13.86 ± 0.00	17.01 ± 0.04	14.75 ± 0.05	18.31 ± 0.02	17.01 ± 0.01	17.09 ± 0.01	18.40 ± 0.00
**11**	Me. C18:1n7	1824	1826	96	0.98 ± 0.01	0.69 ± 0.01	0.98 ± 0.00	1.01 ± 0.01	0.94 ± 0.02	0.96 ± 0.01	1.01 ± 0.00
**12**	Me. C18:2n6 (9Z.12Z)	1860	1851	98	56.11 ± 0.07	56.52 ± 0.28	55.66 ± 0.17	53.56 ± 0.17	54.28 ± 0.42	54.48 ± 0.24	53.30 ± 0.32
**13**	Me. C18:3n6 (6Z.9Z.12Z)	1865	1858	96	2.58 ± 0.03	1.49 ± 0.00	1.98 ± 0.17	1.78 ± 0.18	2.28 ± 0.35	2.21 ± 0.15	2.08 ± 0.17
**14**	Me. C18:3n3 (9Z.12E.15E)	1873	1875	92	0.03 ± 0.00	0.02 ± 0.00	0.02 ± 0.00	0.02 ± 0.00	0.02 ± 0.00	0.03 ± 0.01	0.01 ± 0.00
**15**	Me. C18:3n3 (9Z.12E.15Z)	1883	1880	96	0.05 ± 0.00	0.04 ± 0.00	0.05 ± 0.00	0.05 ± 0.00	0.04 ± 0.00	0.03 ± 0.02	0.04 ± 0.00
**16**	Me. C18:3n3 (9Z.12Z.15Z)	1906	1902	98	13.85 ± 0.00	12.78 ± 0.06	13.77 ± 0.04	12.13 ± 0.01	12.52 ± 0.15	12.38 ± 0.07	11.56 ± 0.09
**17**	Me. C18:4n3 (6Z.9Z.12Z.15Z)	1913	1909	97	0.75 ± 0.00	0.40 ± 0.03	0.61 ± 0.01	0.44 ± 0.00	0.61 ± 0.08	0.58 ± 0.06	0.52 ± 0.08
**18**	Me. C20:0	2002	2000	96	0.89 ± 0.00	0.72 ± 0.00	0.90 ± 0.01	0.90± 0.00	0.85 ± 0.01	0.87 ± 0.01	0.89 ± 0.01
**19**	Me. C20:1n9	2009	2008	92	0.02 ± 0.00	0.02 ± 0.00	0.01 ± 0.00	0.02 ± 0.00	0.04 ± 0.00	0.03 ± 0.01	0.03 ± 0.00
**20**	Me. C20:1n7	2016	2015	98	0.40 ± 0.00	0.33 ± 0.01	0.43 ± 0.00	0.44 ± 0.00	0.44 ± 0.00	0.44 ± 0.00	0.47 ± 0.00
**21**	Me. C20:2n6 (11Z.14Z)	2057	2055	96	0.06 ± 0.00	0.04 ± 0.00	0.06 ± 0.00	0.06 ± 0.00	0.05 ± 0.00	0.05 ± 0.00	0.06 ± 0.00
**22**	Me. C21:0	2103	2102	90	0.02 ± 0.00	0.03 ± 0.02	0.02 ± 0.00	0.02 ± 0.00	0.03 ± 0.01	0.02 ± 0.01	0.02 ± 0.00
**23**	Me. C20:3n3 (11Z.14Z.17Z)	2110	2109	89	0.01 ± 0.00	0.01 ± 0.00	0.01 ± 0.00	0.01 ± 0.00	0.02 ± 0.00	0.01 ± 0.00	0.01 ± 0.00
**24**	Me. C22:0	2201	2200	91	0.37 ± 0.00	0.31 ± 0.00	0.40 ± 0.00	0.42 ± 0.00	0.40 ± 0.00	0.41 ± 0.00	0.45 ± 0.01
**25**	Me. C22:1n9	2219	2217	91	0.03 ± 0.00	0.02 ± 0.00	0.02 ± 0.00	0.03 ± 0.00	0.04 ± 0.00	0.04 ± 0.00	0.07 ± 0.00
**26**	Me. C23:0	2303	2301	95	0.04 ± 0.00	0.03 ± 0.01	0.04 ± 0.00	0.05 ± 0.00	0.04 ± 0.00	0.04 ± 0.00	0.05 ± 0.00
**27**	Me. C24:0	2401	2400	93	0.16 ± 0.00	0.13 ± 0.01	0.18 ± 0.00	0.19 ± 0.00	0.18 ± 0.00	0.19 ± 0.00	0.22 ± 0.00
**28**	Me. C24:1n9	2421	2420	90	0.02 ± 0.00	0.02 ± 0.00	0.02 ± 0.00	0.03 ± 0.00	0.04 ± 0.01	0.03 ± 0.00	0.03 ± 0.00
**29**	Me. C25:0	2496	2494	89	0.01 ± 0.00	0.02 ± 0.01	0.02 ± 0.00	0.02 ± 0.00	0.02 ± 0.01	0.02 ± 0.00	0.02 ± 0.00
**30**	Me. C26:0	2598	2600	91	0.03 ± 0.00	0.02 ± 0.01	0.03 ± 0.01	0.03 ± 0.00	0.03 ± 0.01	0.03 ± 0.00	0.02 ± 0.00
	**SFA**				11.15 ± 0.04	10.51 ± 0.18	11.53 ± 0.05	12.02 ± 0.04	11.59 ± 0.08	11.54 ± 0.05	12.30 ± 0.06
	**MUFA**				15.43 ± 0.02	18.13 ± 0.06	16.33 ± 0.06	19.96 ± 0.03	18.61 ± 0.04	18.70 ± 0.04	20.13 ± 0.02
	**PUFA**				73.49 ± 0.11	71.41 ± 0.38	72.21 ± 0.40	68.07 ± 0.37	69.85 ± 1.00	69.81 ± 0.56	67.63 ± 0.87
	**ω6**				58.78 ± 0.10	58.07 ± 0.28	57.74 ± 0.34	55.42 ± 0.35	56.63 ± 0.78	56.76 ± 0.39	55.46 ± 0.71
	**ω3**				14.69 ± 0.01	13.25 ± 0.09	14.45 ± 0.05	12.64 ± 0.01	13.21 ± 0.22	13.04 ± 0.16	12.14 ± 0.17
	**ω6/ω3**				4.00 ± 0.01	4.38 ± 0.05	4.00 ± 0.04	4.39 ± 0.03	4.29 ± 0.13	4.35 ± 0.09	4.57 ± 0.12
	**SFA/MUFA**				0.72 ± 0.01	0.58 ± 0.01	0.71 ± 0.01	0.60 ± 0.01	0.62 ± 0.01	0.62 ± 0.01	0.61 ± 0.01
	**PUFA/SFA**				6.59 ± 0.04	6.79 ± 0.16	6.26 ± 0.06	5.66 ± 0.05	6.03 ± 0.13	6.05 ± 0.08	5.49 ± 0.10
	**AI**				0.08 ± 0.00	0.07 ± 0.00	0.08 ± 0.00	0.08 ± 0.00	0.08 ± 0.00	0.08 ± 0.00	0.08 ± 0.00
	**TI**				0.12 ± 0.00	0.11 ± 0.01	0.12 ± 0.00	0.13 ± 0.02	0.13 ± 0.00	0.13 ± 0.00	0.14 ± 0.00
	**h/H**				12.86 ± 0.03	13.81 ± 0.16	12.33 ± 0.09	11.73 ± 0.08	12.42 ± 0.16	12.61 ± 0.19	11.45 ± 0.14

^a^ Tentative identification according to the fragmentation pattern and the retention behavior.

**Table 2:** List of identified glycerolipids in the lipid fraction of the hempseed-derived products, along with the PN, total carbon number (CN) and double bonds (DB), experimental (LRI_exp_) and theoretical LRI (LRI_tab_) values and their difference (Δ) and relative content (%).

PN	Compound	CN	DB	LRI_exp_	LRI_tab_^a^	Δ	Oil I	Oil II	Oil III	Oil IV	Flour II	Flour IV	Residue IV
24	LnLn	36	6	2608 ^#^	-	-	0.67 ± 0.04	0.27 ± 0.01	0.88 ± 0.01	0.68 ± 0.04	1.16 ± 0.04	1.37 ± 0.09	0.54 ± 0.01
26	LLn	36	5	2764	-	-	1.00 ± 0.06	1.04 ± 0.04	1.6 ± 0.02	0.57 ± 0.03	2.83 ± 0.08	3.59 ± 0.23	1.71 ± 0.00
26	OSt	36	5	2781	-	-	0.15 ± 0.01	0.18 ± 0.02	0.26 ± 0.01	0.14 ± 0	0.66 ± 0.02	0.97 ± 0.11	0.46 ± 0.04
28	LL	36	4	2895	-	-	1.53 ± 0.15	1.92 ± 0.05	2.02 ± 0.13	0.79 ± 0.02	4.23 ± 0.1	5.29 ± 0.07	2.79 ± 0.04
28	OLn + LnP	36/34	4/3	2917	-	-	0.3 ± 0.02	0.35 ± 0.01	0.46 ± 0.05	0.37 ± 0.01	0.91 ± 0.09	1.11 ± 0.1	0.82 ± 0.02
30	LO + LP	36/34	3/2	3100	-	-	0.54 ± 0.10	0.34 ± 0.06	0.51 ± 0.02	0.32 ± 0.01	1.13 ± 0.08	1.29 ± 0.15	0.94 ± 0.12
36	LnLnLn	54	9	3675 *	3668	7	0.87 ± 0.02	1.14 ± 0.05	1.08 ± 0.04	0.89 ± 0.01	0.86 ± 0.06	1.37 ± 0.16	0.83 ± 0.04
36	LLnSt + γLnLnLn	54	9	3703	-	-	1.14 ± 0.05	1.08 ± 0.03	1.14 ± 0.05	0.66 ± 0.08	0.82 ± 0.07	0.82 ± 0.06	0.55 ± 0.01
36	γLnγLnLn	54	9	3720	-	-	0.13 ± 0.01	0.26 ± 0.02	0.22 ± 0.01	0.34 ± 0.04	-	-	-
38	LnLLn + γLnLLn	54	8	3845 *	3830/-	15/-	6.95 ± 0.27	6.94 ± 0.20	7.42 ± 0.16	5.84 ± 0.22	6.27 ± 0.26	5.97 ± 0.28	6.29 ± 0.00
38	γLnγLnL + LLSt	54	8	3876 *	3867/3890 ^b^	9/14	3.89 ± 0.03	2.61 ± 0.00	3.42 ± 0.05	2.29 ± 0.01	2.62 ± 0.05	2.69 ± 0.25	2.54 ± 0.04
40	LLLn + LLγLn + LnOLn	54	7	4008	3993/3999/4011	15/9/3	17.53 ± 0.49	15.66 ± 0.49	16.96 ± 0.53	16.60 ± 0.33	14.57 ± 0.76	15.65 ± 1.06	14.90 ± 0.11
40	OLSt + LnLnP + LnOγLn	54/52/54	7/6/7	4036 *	-/4023/-	-/13/-	4.38 ± 0.13	2.98 ± 0.05	4.21 ± 0.3	2.93 ± 0.05	2.62 ± 0.32	2.97 ± 0.04	2.67 ± 0.01
40	γLnLnP + StLP	52	6	4048 *	-/4049 ^b^	-	0.46 ± 0.06	0.50 ± 0.05	0.68 ± 0.14	0.42 ± 0.07	0.34 ± 0.02	0.42 ± 0.03	0.36 ± 0.07
42	LLL	54	6	4165 *	4160	5	20.31 ± 0.3	19.35 ± 0.21	19.86 ± 0.5	20.11 ± 0.13	18.31 ± 0.22	17.12 ± 0.37	19.18 ± 0.01
42	OLLn	54	6	4191	4192	−1	5.56 ± 0.14	5.97 ± 0.04	5.04 ± 0.04	5.98 ± 0.35	5.56 ± 0.19	5.74 ± 0.20	5.79 ± 0.09
42	OLγLn+ LnLP	54/52	6/5	4209	4196/4217	13/−8	4.95 ± 0.15	4.54 ± 0.38	4.41 ± 0.34	4.88 ± 0.21	4.37 ± 0.18	3.90 ± 0.08	4.63 ± 0.13
42	SLnLn + γLnLP	54/52	6/5	4235	4216/-/4221/4221	19/-/14/14	1.19 ± 0.03	0.82 ± 0.01	0.94 ± 0.18	0.96 ± 0.14	0.91 ± 0.06	0.93 ± 0.08	1.02 ± 0.02
44	OLL	54	5	4348	4342	6	8.57 ± 0.16	9.76 ± 0.08	8.52 ± 0.43	11.13 ± 0.32	9.72 ± 0.42	9.55 ± 0.20	10.18 ± 0.14
44	LLP + OOLn	52/54	4/5	4369	4358/4360	11/9	6.84 ± 0.18	7.15 ± 0.03	6.41 ± 0.60	7.97 ± 0.12	6.86 ± 0.05	6.18 ± 0.21	7.69 ± 0.12
44	SLLn + LnOP	54/52	5/4	4396	4378/4383	18/13	2.15 ± 0.12	2.34 ± 0.07	1.99 ± 0.29	2.36 ± 0.13	2.36 ± 0.08	2.04 ± 0.08	2.05 ± 0.07
44	SLγLn + γLnOP	54/52	5/4	4420	-/4403	-/17	0.48 ± 0.07	0.40 ± 0.01	0.42 ± 0.07	0.38 ± 0.05	0.57 ± 0.12	0.40 ± 0.02	0.31 ± 0.02
46	C20:1LL	56	5	4502	4512	−10	2.93 ± 0.23	4.24 ± 0.07	3.16 ± 0.19	4.14 ± 0.23	3.71 ± 0.28	3.66 ± 0.19	4.1 ± 0.05
46	OOL	54	4	4537	4522	15							
46	OLP + SLL	52/54	3/4	4562	4548/4548	14/14	3.63 ± 0.07	4.64 ± 0.1	3.7 ± 0.04	4.26 ± 0.44	4.09 ± 0.37	3.8 ± 0.23	4.19 ± 0.03
46	ALLn + SOLn + PLP	56/54/50	5/4/2	4586	-/4575/4571	/11/15	0.69 ± 0.09	0.84 ± 0.02	0.61 ± 0.04	0.72 ± 0.08	0.78 ± 0.03	0.73 ± 0.06	0.74 ± 0.07
48	C20:1LO	56	4	4728	4708	20	0.11 ± 0.01	0.16 ± 0	0.22 ± 0.03	0.13 ± 0	0.18 ± 0.02	0.12 ± 0.01	0.24 ± 0.02
48	OOO	54	3	4740	4729	11	0.24 ± 0.05	0.49 ± 0.04	0.39 ± 0	0.51 ± 0.02	0.4 ± 0.03	0.35 ± 0.07	0.56 ± 0.04
48	ALL + SOL + OOP	56/54/52	4/3/2	4761	-/4746/4756	-/15/5	1.41 ± 0.29	2.03 ± 0.13	1.63 ± 0.07	1.95 ± 0.07	1.81 ± 0.07	1.66 ± 0.36	2.26 ± 0.28
48	BLLn + AOLn	58/56	5/4	4777	-	-	0.19 ± 0.01	0.23 ± 0.02	0.25 ± 0				
48	SLP + POP	52/50	2/1	4789	-/4776	-/13	0.04 ± 0.01	0.06 ± 0	0.06 ± 0.01				
50	BLL+ ALO +LgLLn+ SOO	58/56/60/54	4/3/5/2	4948	-/-/-/4948	-/-/-/0	0.91 ± 0.03	1.24 ± 0.05	1.09 ± 0.02	1.23 ± 0.03	1 ± 0.16	1.08 ± 0.1	1.23 ± 0.01
52	LgLL + BLO + AOO	60/58/56	4/3/2	5126	-/-/5138 ^c^	-/-/-12	0.24 ± 0	0.38 ± 0	0.36 ± 0.02	0.43 ± 0.05	0.35 ± 0.06	0.34 ± 0.03	0.44 ± 0
54	LgLO + BOO	60/58	3/2	5336	-	-	0.05 ± 0.00	0.08 ± 0.01	0.08 ± 0.01				

^a^ LRI theoretical values according to Rigano et al. [19]; ^b^ LRI theoretical values according to Rigano et al. [18]. ^c^ LRI theoretical values according to Oteri et al. [43]; * unambiguously identified based only on the LRI criterion; # extrapolated value. Fatty acid abbreviations: Ln: linolenic acid (C18:3); L: linoleic acid (C18:2); O: oleic acid (C18:1); St: stearidonic acid (C18:4); P: palmitic acid (C16:0); S: stearic acid (C18:0); A: arachidic acid (C20:0); B:behenic acid (C22:0); and Lg: lignoceric acid (C24:0).

## Data Availability

The authors declare that all data supporting the findings of this study are available within the article.

## Sample Availability

Samples of the compounds areavailable from the authors.
